# Small Molecule Inhibitors Target the Tissue Transglutaminase and Fibronectin Interaction

**DOI:** 10.1371/journal.pone.0089285

**Published:** 2014-02-20

**Authors:** Bakhtiyor Yakubov, Lan Chen, Alexey M. Belkin, Sheng Zhang, Bhadrani Chelladurai, Zhong-Yin Zhang, Daniela Matei

**Affiliations:** 1 Indiana University Department of Medicine, Indiana University School of Medicine, Indianapolis, Indiana, United States of America; 2 Chemical Genomics Core Facility, Indiana University School of Medicine, Indianapolis, Indiana, United States of America; 3 University of Maryland Department of Biochemistry and Molecular Biology, Indiana University School of Medicine, Indianapolis, Indiana, United States of America; 4 Indiana University Simon Cancer Center, Indiana University School of Medicine, Indianapolis, Indiana, United States of America; 5 Department of Biochemistry and Molecular Biology, Indiana University School of Medicine, Indianapolis, Indiana, United States of America; 6 VA Roudebush Hospital, Indiana University School of Medicine, Indianapolis, Indiana, United States of America; Seoul National University, Republic of Korea

## Abstract

Tissue transglutaminase (TG2) mediates protein crosslinking through generation of ε−(γ-glutamyl) lysine isopeptide bonds and promotes cell adhesion through interaction with fibronectin (FN) and integrins. Cell adhesion to the peritoneal matrix regulated by TG2 facilitates ovarian cancer dissemination. Therefore, disruption of the TG2-FN complex by small molecules may inhibit cell adhesion and metastasis. A novel high throughput screening (HTS) assay based on AlphaLISA™ technology was developed to measure the formation of a complex between His-TG2 and the biotinylated FN fragment that binds TG2 and to discover small molecules that inhibit this protein-protein interaction. Several hits were identified from 10,000 compounds screened. The top candidates selected based on >70% inhibition of the TG2/FN complex formation were confirmed by using ELISA and bioassays measuring cell adhesion, migration, invasion, and proliferation. In conclusion, the AlphaLISA bead format assay measuring the TG2-FN interaction is robust and suitable for HTS of small molecules. One compound identified from the screen (TG53) potently inhibited ovarian cancer cell adhesion to FN, cell migration, and invasion and could be further developed as a potential inhibitor for ovarian cancer dissemination.

## Introduction

Protein-protein interactions (PPIs) regulate numerous cellular functions, including cell interactions with the extracellular matrix (ECM) and signaling pathways that go awry in cancer. Therefore, disruption of PPIs has been a desirable goal for drug discovery in cancer, as well as in other pathological conditions. The classical approach consists of designing peptides or peptide mimetics that competitively inhibit specific PPIs. Peptides inhibitors have been useful to demonstrate proof of principle concepts related to biological processes regulated by PPIs; however their restricted bioavailability and stability has limited their usefulness for clinical development. Small molecule inhibitors (SMIs) offer several advantages. They are fast-acting, reversible, and can serve as leads for subsequent drug optimization efforts. In this manuscript, we used high throughput screening (HTS) to identify SMIs for interacting tissue transglutaminase (TG2) and fibronectin (FN).

TG2 is a member of the transglutaminase family that catalyzes Ca^2+^ dependent protein crosslinking via formation of amide bonds. One of its unique properties compared to the other transglutaminases is its interaction with FN. The FN-binding site of TG2 has been mapped to amino acids 88–106 at its N-terminus [1], encompassing two anti-parallel β-strands located within the first β sandwich domain of TG2 and forming an extended hairpin. This region binds with high affinity to the 42-kDa domain of FN, consisting of modules I_6_ II_1,2_ I_7–9_ [1]–[3]. The TG2-FN interaction strengthens β-integrin-mediated cellular adhesion to the ECM [4], playing a role in a variety of physiological and pathological processes. The well-described recognition sequence for FN on TG2 provides an opportunity for developing SMIs to disrupt this interaction. Often PPIs comprise large and flat interfaces difficult to block by SMIs; however, the TG2-FN interaction is an attractive target, because the interacting domains are not flat surfaces, but rather a relatively small TG2 hairpin inserting into a deep pocket of FN.

We and others described increased expression of TG2 in epithelial malignancies, specifically in ovarian, breast and pancreatic cancers [5]–[7]. TG2 has been linked to various functions in this context, but by and large the protein acts as a promoter of chemotherapy resistance [8], [9] and a facilitator of metastasis [5], [10], [11]. By using intraperitoneal and orthotopic ovarian cancer xenograft models, our group demonstrated that TG2 increases peritoneal metastasis [5], [11] and linked this process to β-integrin mediated ovarian cancer cell adhesion to the peritoneal matrix. We also showed that TG2 induces epithelial-to-mesenchymal transition (EMT) [11] which is a critical step in the initiation of metastasis and that the FN-binding domain of TG2 is sufficient to initiate this process [12], [13].

In addition, the TG2-mediated interaction between β-integrin and FN activates cell survival pathways [14] and contributes to doxorubicin resistance in breast cancer cells [15], as well as cisplatin and dararbazine resistance in melanoma cells [16]. Downregulation of TG2 in U87MG glioblastoma cells disrupted the assembly of FN in the ECM and sensitized tumors to chemotherapy [17], supporting the key role of this protein at the interface between cancer cells and the surrounding ECM. These findings support the concept that targeting the TG2-FN interaction with SMIs will disrupt cancer cell adhesion to the ECM, and subsequently inhibit initiation of metastasis and development of drug resistance.

In this study, we used HTS technology to identify SMIs for the TG2-FN complex. For this, we developed and optimized an AlphaLISA^TM^ assay to measure the interaction between the two proteins and to screen a 10,000 compounds library for potential inhibitors. The ChemDiv collection used for this study contains highly purified compounds, diverse in structure, with drug-like physical and chemical properties. The compounds obey the Lipinski’s “rule of five” demonstrating good ADME (absorption, distribution, metabolism and exertion) profiles, rendering them suitable compounds for future development. Through subsequent cell based validation assays we identified several hits that potently blocked TG2-mediated cell adhesion and migration. We propose that these SMIs can be further optimized and studied as potential inhibitors of metastasis.

## Materials and Methods

### Cells

SKOV3 and IGROV1 cells were obtained from the American Type Culture Collection (ATCC, Manassas, VA), and cultured in growth media containing 1∶1 MCDB 105 (Sigma, St. Louis, MO) and M199 (Cellgro, Manassas, VA) in the presence of 10% FBS at 37°C in a humidified atmosphere containing 5% CO_2_.

### Chemical Compounds

A library of 10,000 compounds with diverse structures was obtained from the Chemical Genomics Core Facility at Indiana University. These 10,000 compounds were selected from over 500,000 novel compounds synthesized at ChemDiv in recent years, representing a large chemical space. Average purity of the compounds measured by mass spectrometry (MS) was greater than 95% (Figures S1 and S2 in File S1). Identified in the primary screen and confirmed in a re-screen, 77 compounds were purchased from ChemDiv, Inc (San Diego, CA).

### HPLC-MS Analysis of Compound TG53

Analytical HPLC-MS analysis was carried out on a Agilent 1200 analytic HPLC system with a 6130 Quadrupole MS detector, equipped with a Phenomenex Kinetex 2.6 μ XB-C18 column (2.5 μm, 4.6 mm × 50 mm), eluted with a 0–90% MeOH-H_2_O with 0.1% (v/v) TFA at 0.8 mL/min flow-rate (gradient method: 3 min 0% MeOH, followed by 9 min 0–90% MeOH linear gradient, followed by 5 min 90% MeOH).

### Protein Purification

Full length TG2 was expressed in *Escherichia coli* (E. coli) and purified as described [18], with minor modifications. Briefly, overnight cultures from *E. coli* BL21 cells (Invitrogen, Grand Island, NY) transformed with pET28-TG2 expression vector (gift from Dr. R. Cerione Cornell University) were grown at 37°C. Protein expression was induced with 300 uM IPTG for 24 h at 25°C. Cell pellets were lysed by sonication at 0°C in lysis buffer (50 mM Na_2_HPO_4_, pH 7.5, 400 mM NaCl, 5 mM benzamidine, 5 mM 2-mercaptoethanol) containing 50 μM GTP, 50 μM ATP, and 50 μg/ml PMSF. After sonication, NP-40 was added to a concentration of 0.5% (vol/vol). Cell debris was removed by high-speed centrifugation and the supernatant was passed through a Talon metal-affinity resin column (Clontech, Mountain View, CA). The His_6_-TG2 fusion protein was eluted with 50 mM Hepes (pH 7.0), 50 mM NaCl, 5 mM 2-mercaptoethanol, 20 μM GDP, 160 mM imidazole. The eluted protein was dialyzed against 50 mM MES (pH 6.5), 50 mM NaCl, 10% glycerol, 1 mM EDTA, 5 mM DTT and loaded onto a HiTrap anion exchange column (GE Healthcare Life Biosciences, Pittsburgh, PA). Recombinant TG2 was eluted by using a gradient of 50 mM to 450 mM NaCl in MES buffer. Fractions containing TG2 were pooled and concentrated. Purity of TG2 was confirmed by SDS-PAGE. The biotinylated 42-kDa gelatin binding fragment of FN (FN42) was obtained by digestion with thermolysin from human plasma, as previously described [1], [14], [19]. Briefly, the thermolysin digest of human plasma FN was applied on a column with Gelatin-Sepharose (Sigma-Aldrich, St. Louis, MO) and bound material containing the 42 kDa (modules I_6_II_1,2_I_7–9_) and the 56 kDa (modules I_6_II_1,2_III_1_) fragments was eluted with 1M arginine in 20 mM TrisHCl ph7.5. The resulting gelatin-binding fragments were separated by binding to Heparin-Sepharose as only the 56 kDa fragment was able to bind under low salt conditions [19], whereas the FN42 fragment remained in the flow through. The FN42 fragment was biotinylated using sulfo-NHS-biotin (Pierce, Rockfort, IL) and separated from free label by dialysis. The labeling efficiency was 4.8 biotin groups per FN42 molecule.

### AlphaLISA Assay for TG2-FN Interaction

An AlphaLISA assay in microplate format (PerkinElmer, Shelton, CT) was developed to measure the TG2-FN interaction. Interacting labeled proteins bring donor and acceptor beads into close proximity (< 200 nm) generating a measurable chemiluminescence signal. In brief, biotinylated FN42 and His_6_-TG2 were serially diluted in 1.25X Universal Buffer (PerkinElmer) at concentrations ranging from 1 to 10 nM and 1 to 25 nM, respectively, in 96–well white solid plates (PerkinElmer). After 1 hour incubation at RT, 10 μl nickel chelate acceptor beads (20 μg/ml) were added and incubated with the protein mixture for 1 h at RT. Lastly 10 μl streptavidin donor beads (20 μg/ml) were added before the reading. Nonspecific binding was measured in the absence of TG2 representing less than 5% of total binding. The hook point was calculated from the titration curve plotted using GraphPad Prism software (San Diego, CA). For the subsequent high throughput screening, FN42 was diluted to 2.8 nM and TG2 to 8.3 nM, correspondingly, to achieve optimal binding affinity. To determine the dynamic range of the assay the Z′ value was measured by taking into account the means (µ) and the standard deviations (σ) of the positive and the negative control, according to the formula shown [20]. The controls represent the upper and lower limit of the assay
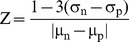



Average alpha signal for 1% DMSO alone was 105757.4 ± 9995.5 (mean ± SD) and estimated Z′ factor was 0.7, indicating robust assay for HTS. The primary screen was performed in 384-well plates with compounds arrayed at 10 µΜ concentration in 1% DMSO. The alpha signal was measured using EnVision® Multilabel Reader (PerkinElmer). After the initial screening, compounds inducing greater than 50% of inhibition were re-tested for validation.

### Enzyme-linked Immunosorbent Assay (ELISA)

ELISA measured the TG2-FN interaction in 96 well plates pre-coated with monoclonal anti-His antibody (Roche, Indianapolis, IN; 1∶6000). Protein mixture of 10 nM TG2 and 3 nM FN42 was used and SMIs were added at 1–30 μM concentrations. After 1 h incubation at RT, plates were washed 3 times with 0.5% BSA in PBS prior to 1 h incubation with streptavidin-HRP (Cell Signalling, Boston, MA) diluted in PBS (1∶2000). Following four washes, 3,3′,5,5′ tetramethylbenzidine (TMB) substrate (Sigma) was added for 5–15 minutes and the optical density was determined at 405 nM with 450 nm as a reference filter using Ultra Multifunctional Microplate Reader (Tecan, Durham, NC). Data shown are means ± SE of three replicates. The IC50 for TG53 was calculated after incubating TG2 and FN42 in the presence of various concentrations of TG53 (from 0 μM to 16 μM). The data were fitted by using non-linear regression to a 1-site competition model by GraphPad Prism. The inhibition constant Ki was computed by using equations KmObs = Km*(1+[I]/Ki) and Y = Vmax*X/(KmObs+X) in GraphPad Prism, where **X** is substrate concentration, **Y** is response, **I** is the concentration of inhibitor, **Vmax** is the maximum response, in the absence of inhibitor, expressed in the same units as Y. **Km** is the Michaelis-Menten constant, expressed in the same units as X.

### Solid-phase Adhesion Assays

Cells were labeled with calcein AM (Molecular Probes) and seeded at a density of 4×10^4^ cells in 96-well plates pre-coated with FN (5 μg/mL, Sigma), collagen type I (10 μg/ml, Sigma), or bovine serum albumin (BSA, 1% w/v, Sigma) in the presence of selected compounds diluted from 1–25 μM. All matrices were blocked with BSA (1%) for 1 hour prior to cell seeding. After 60 minutes, adherent cells were quantified in a fluorescence plate reader (Applied Biosystems). Experiments were performed in quadruplicate and repeated at least twice.

### Cell Proliferation

Cells were seeded in 96-well plates at a density of 2×10^4^ cells/well in medium containing 10% FBS. After 16 h cells were allowed to proliferate in serum free medium for up to 48 h in presence or absence of selected SMIs. Cell proliferation was measured by using the Cell Counting Kit-8 (CCK-8, Dojindo, Japan) following the manufacturer’s directions. Absorbance values were read using the Ultra Multifunctional Microplate Reader (Tecan, Durham, NC). EC_50_ was determined by nonlinear regression using GraphPad Prism software (GraphPad Software, Inc., La Jolla, CA).

### Scratch Wound Healing Assay

Wound healing was performed in 12-well plates coated with 1 μg/ml FN (Sigma) overnight at 4°C. SKOV3 cells were plated at a density of 2×10^5^ cells/well in media containing 2% FBS. Under these conditions cells adhered on substrate, but cell proliferation was minimized. After 16 hours the cell monolayer was scraped in straight line. Cell debris was removed and serum free media containing selected SMIs or control was replenished. For consistency during image acquisition, a reference point was marked close to the scratch mark and the plate was imaged under phase-contrast microscope at 0, 2, 6, 12 and 24 hours. Wound closure was assessed as the distance between the wound edges, calculated and quantified using the Image Pro-Express software. The percentage of wound closure was evaluated using the formula (wound size at 24 hours/ initial wound size) × 100. The rate of migration (μm/h) of SKOV3 cells into the wound space was evaluated as the distance difference between wound edges at 0 and 24 h, divided by 24. The experiments were performed in duplicates.

### Invasion Assay

After serum starvation overnight**,** 10^6^ SKOV3 cells were seeded into Millicell Cell Culture Inserts (Millipore, Billerica, MA) coated with 0.3 mg/ml of matrigel (BD Biosciences, San Jose, California) in serum free media containing selected SMIs or controls (10 μM). Media in the bottom part of the transwell contained 20% FBS. After 24 h incubation at 37°C and 5% CO_2_ transwells were washed, migrating cells were fixed with 10% formalin (Fisher Scientific, Waltham, MA) and stained for 30 minutes with 0.2% crystal violet solution (MP Biomedicals, Solon, OH). Washed and dried inserts were then photographed and the number of stained cells was counted in 5 random fields. Average numbers of invading cells per insert area were calculated and reported relative to the number of cells seeded. The experiment was performed in duplicates.

### TG2 Enzymatic Activity Assay

To measure transglutaminase enzymatic activity, the formation of hydroxamate from Nα-CBZ-glutaminyl-glycine and hydroxylamine was measured by using a colorimetric assay previously described [21]. In brief, 1 µg of purified His-TG2 was incubated with Nα-CBZ-glutaminyl-glycine and hydroxylamine in the presence of Ca^2+^ at 37°C and TGase activity was measured as the amount of hydroxamate generated in the reaction. The colorimetric reaction was measured at 525 nm in an ELISA plate reader (SpectraMax 190). Selected inhibitors were used in concentrations ranging from 1 to 30 µM. All assays were performed in triplicates. Data are presented as means +/− SD.

## Results

### AlphaLISA Assay Measures TG2-FN Interaction

**Figure 1:**
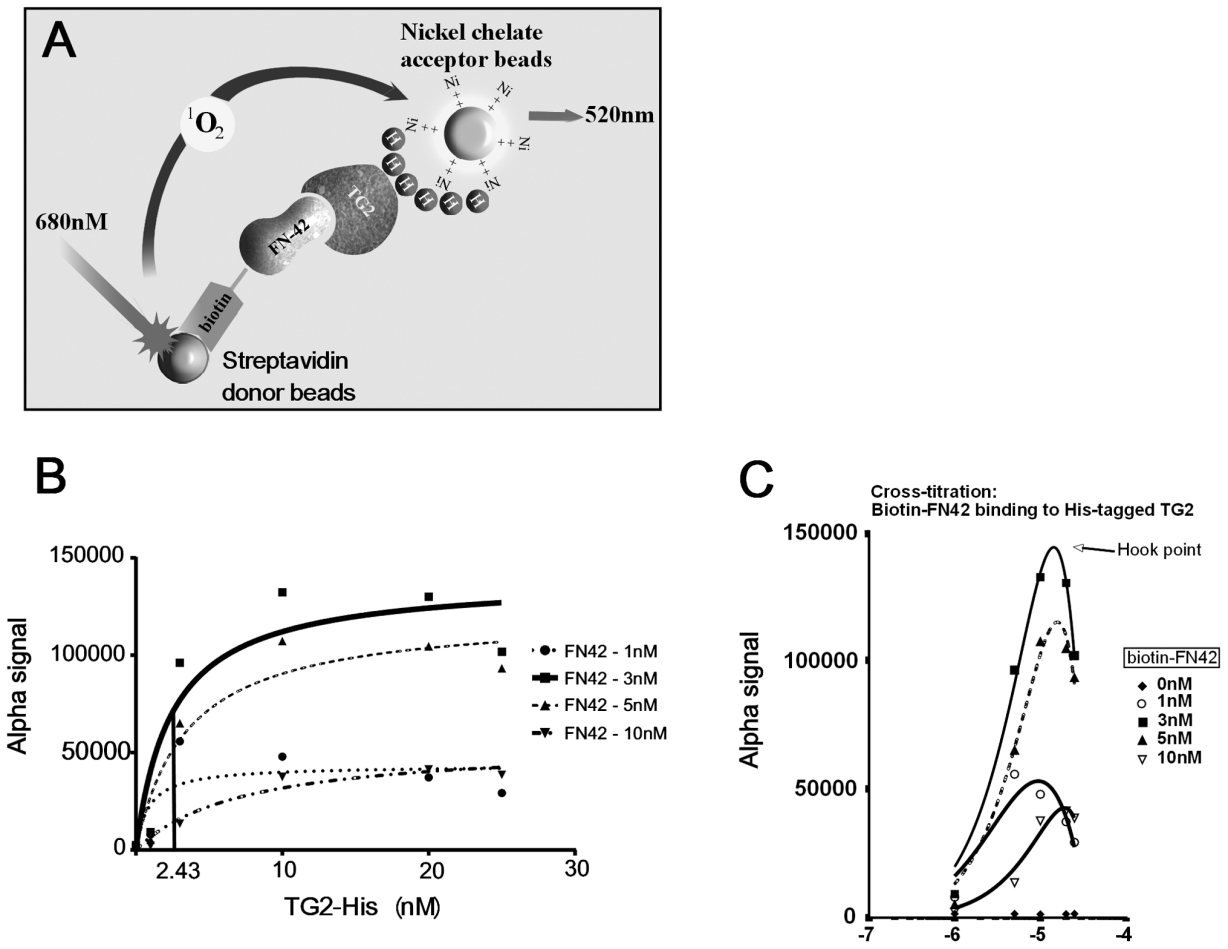
Characteristics of the AlphaLISA^TM^ assay developed to measure the TG2-FN42 interaction. A, Design of the AlphaLISA assay that measures the TG2-FN interaction. Donor beads coated with streptavidin and acceptor nickel chelate beads were used to capture biotinylated FN42 and His tagged TG2 protein, respectively. After excitation at 680 nm singlet oxygen is transferred from donor to acceptor beads coming within a distance of 200 nm, resulting in a chemiluminescence signal. B, Cross-titration was performed to optimize detection of the TG2-FN interaction by the assay. Saturation isotherms of FN42 binding to TG2 were generated. The K_d_ was 2.43 nM. C, Titration curves represented with GraphPad Prism demonstrate reaching the hook point at 3 nM biotinylated FN42 and 10 nM TG2-His.

An AlphaLISA assay adaptable to HTS in 384 well plates was designed to measure the TG2-FN interaction. The assay measures the interaction between His_6_-TG2 bound to Ni-chelated acceptor beads and biotinylated FN42 bound to streptavidin coated donor beads (Figure 1A). Protein concentrations were titrated to optimize the assay, as the alpha beads have specific binding capacity and become progressively saturated at increasing protein concentrations. To determine optimal conditions, beads were incubated with serial concentrations of TG2 and FN42, and saturation curves determined the K_d_ for the interaction. Saturation was reached with 3 nM of FN42 and 10 nM of His_6_-TG2, the K_d_ value being 2.43 nM (Figure 1B). At hook point, both beads are saturated generating the maximum signal. Excess protein above these levels inhibits the association between target molecules and beads causing a decrease in signal. The hook point was reached at 10 nM of His_6_-TG2 and 3 nM of FN42 (Figure 1C). To further evaluate the performance of the assay in HTS mode, the Z′ value was calculated by taking into account the means (µ) and the standard deviations (σ) of the positive and the negative controls. The average Alpha signal was 105757.4 ± 9995.5 (mean ± SD) and the estimated Z′ was 0.7, indicating robustness of the assay for HTS.

### HTS Using the ChemDiv Library

**Figure 2:**
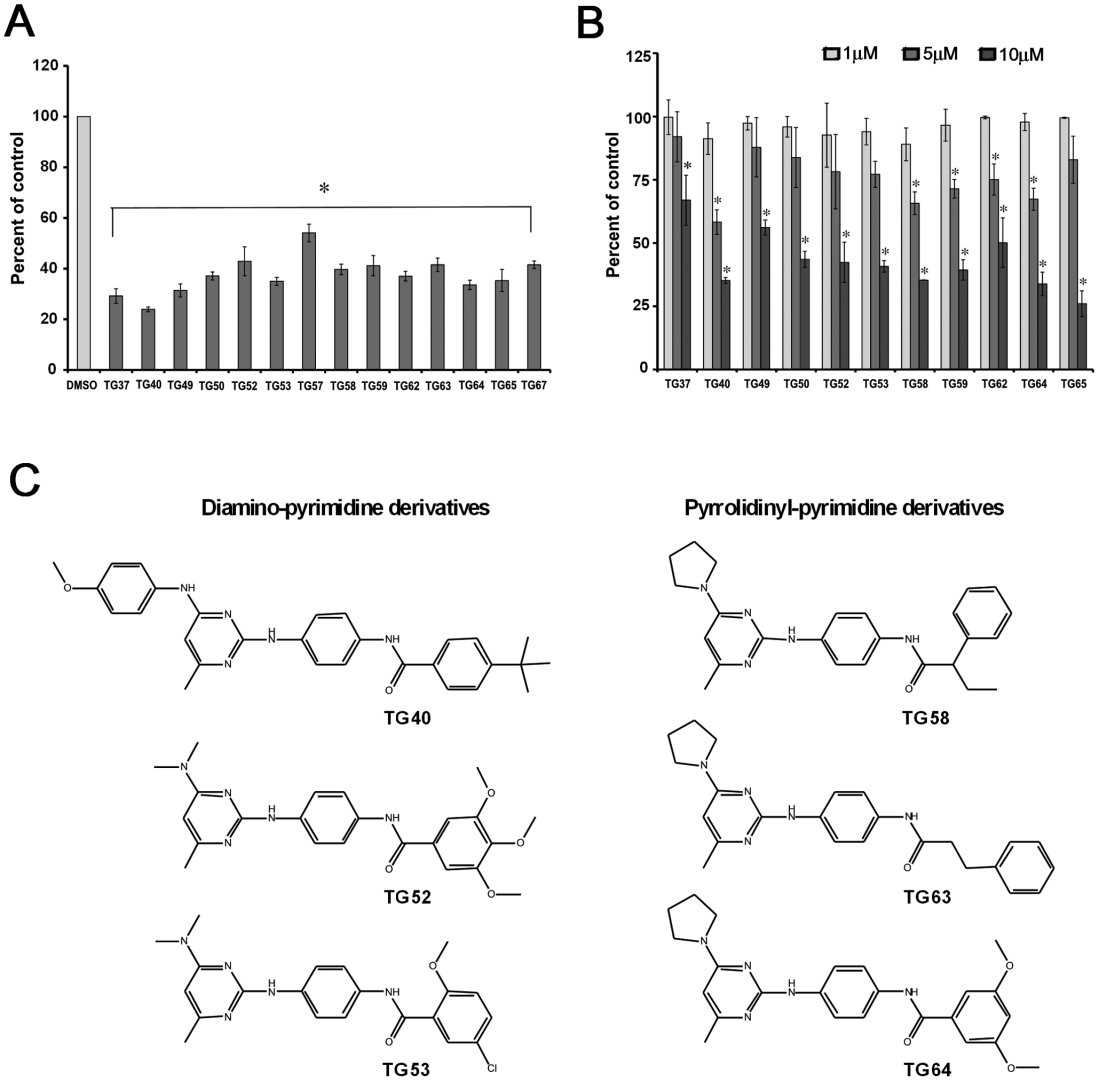
The AlphaLISA assay is used to screen the ChemDiv library. A, The assay identifies SMIs that inhibit the TG2-FN42 interaction by >50% at 10 μM. B, Dose dependent inhibition of TG2-FN42 interaction by selected compounds. Bars represent means +/− SD of triplicate measurements. Asterisks denote *p <*0.05. C, Selected compounds are grouped in two categories based on chemical structure: diamino-pyrimidines and pyrolydinyl-pyrimidines.

The ChemDiv library containing 10,000 diverse compounds arrayed at 10 μM each was screened using the assay described. Primary hits (n = 90) were selected as those compounds yielding ≥ 50% inhibition of control signal and were re-tested. Seventy seven confirmed hits were selected and re-tested in triplicates (Figure 2A). Of those, 14 compounds were selected based on confirmed ≥70% inhibitory effect in validation AlphaLISA assay and were characterized further. Dose-response experiments for selected SMIs using the AlphaLISA assay demonstrated inhibition of the TG2-FN interaction at concentrations greater than 1 μM. The interaction was inhibited by >25% at 5 μM by several SMI (TG49, TG53, TG58, TG59, TG62, TG63, TG64) and by 33% to 79% by all tested compounds at 10 μM concentration (Figure 2B). The top 14 hits have a common core (N2(4-aminophenyl)pyrimidine-2,4-diamine) and belong to two groups based on chemical structures (Table 1). One group of diamino-pyrimidines includes compounds TG37, TG40, TG49, TG50, TG52, TG53 and the other group of pyrolydinyl-pyrimidines encompasses compounds TG58, TG59, TG63, TG64, and TG65 (Figure 2C). MS spectra of selected compounds are included in Figures S1 and S2 in File S1. Purity of compound TG53 was validated by HPLC-MS and was > 98% (Figure S1 in File S1).

**Table 1:** List of top hits from the HTS using AlphaLISA and their predicted physico-chemical characteristics.

	Mass	Formula	LogP	Rule of 5	Bio-availability	Ghose filter	Muegge filter	Veber filter	% inhibitionalphaLISA
**TG 37**	399.4	C23H21N5O2	5	yes	yes	yes	yes	yes	70.8
**TG 40**	481.6	C29H31N5O2	6.8	no	yes	no	no	yes	76.1
**TG 49**	453.5	C27H27N5O2	6.3	no	yes	no	no	yes	68.6
**TG 50**	453.5	C27H27N5O2	6.3	no	yes	no	no	yes	62.9
**TG 52**	437.5	C23H27N5O4	3.6	yes	yes	yes	yes	yes	57.1
**TG 53**	411.9	C21H22ClN5O2	4.5	yes	yes	yes	yes	yes	65
**TG 58**	433.5	C24H27N5O3	4.18	yes	yes	yes	yes	yes	60.3
**TG 59**	463.5	C25H29N5O4	4.03	yes	yes	no	yes	yes	58.8
**TG 62**	425.9	C22H21ClFO	5.25	no	yes	yes	no	yes	63
**TG 63**	415.5	C25H29N5O	5.47	no	yes	yes	no	yes	58.5
**TG 64**	401.5	C24H27N5O	4.92	yes	yes	yes	yes	yes	66.4
**TG65**	415.5	C25H29N5O	5.37	no	yes	yes	no	yes	64.7

### Confirmation of Hits Using ELISA

**Figure 3:**
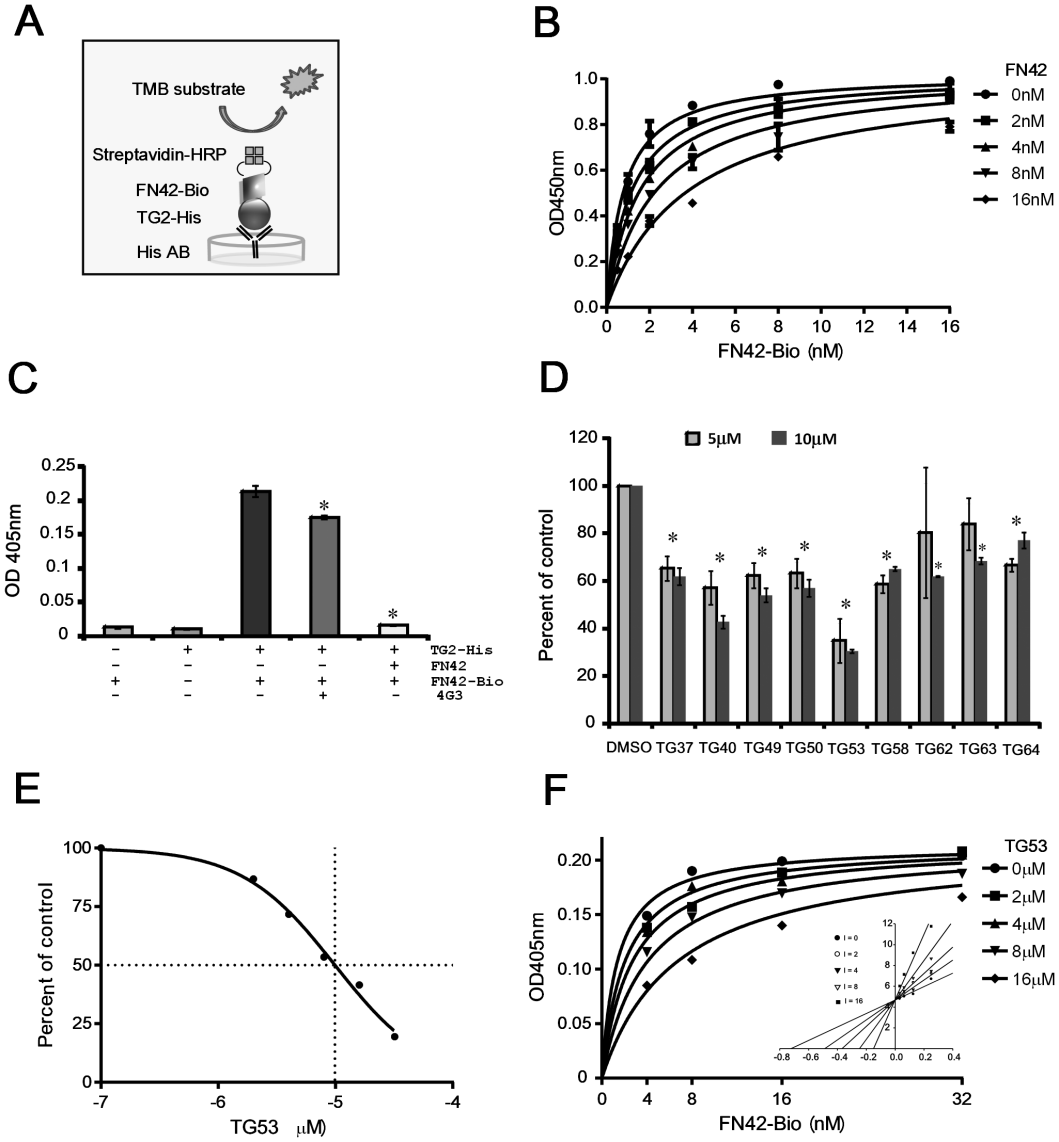
ELISA-based approach measures the TG2-FN interaction. **A**, Design of the ELISA measuring the TG2-FN interaction. His tagged TG2 is captured by the anti-His antibody coating the wells. Biotinylated FN42 interacting with TG2 is recognized by streptavidin-HRP, which reacts with a TMB substrate. The signal is abrogated if the TG2-FN interaction is disrupted. **B**, Specificity of the assay is demonstrated by incubating His-tagged TG2 with increasing concentrations of biotinylated FN42 (from 0 nM to 16 nM) in the presence of unlabeled FN42 (from 0 nM to 16 nM). **C**, ELISA in the presence of the competitive inhibitor 4G3, an anti-TG2 antibody against the FN-binding domain of TG2 (5 μg/ml), and in the presence of unlabeled FN42 (3 nM). **D**, ELISA measures inhibition of the TG2-FN42 interaction by SMIs selected from the AlphaLISA HTS. Bars represent means +/− SD of triplicate measurements. Asterisks denote *p <*0.05. **E**, ELISA measures dose dependent inhibition of TG2-FN42 interaction by TG53. **F**, Saturation curves of FN42 in the presence of increasing concentrations of TG53 were used to calculate the Ki (4.15 μM) of TG53 for TG2. Inset corresponds to representative Lineweaver-Burk plots showing that TG53 competes for the same binding site in TG2 as FN42.

ELISA measured TG2-FN interaction by using biotinylated FN42 and His_6_-TG2 (Figure 3A). Specificity of the assay was confirmed by using increasing concentrations of un-labeled FN42 (from 0 nM to 16 nM) added to the mixture of His-TG2 (5 nM) and biotinylated FN42 (0 nM to 16 nM; Figure 3B). The data indicate a competitive antagonism of unlabeled FN42-Bio towards biotinylated FN42, showing that FN42 is capable of displacing biotinylated FN42 from its binding site in TG2. Additionally, an antibody against the FN binding domain of TG2 (4G3, MAB3839) known to partially block the TG2-FN interaction decreased the ELISA signal by 25%, suggesting that the assay can effectively measure the properties of TG2-FN inhibitors (Figure 3C). Compounds selected from the AlphaLISA screen were validated by ELISA (Figure 3D). Most active compounds inhibited the ELISA signal by 30 to 70%, TG53 being the most active inhibitor. A dose-dependent decrease in the ELISA signal induced by TG53 was used to calculate the IC50 at 10 μM (Figure 3E). The inhibition constant Ki for TG53 was calculated as 4.15 μM from the effect of TG53 on FN42-Bio to TG2 (Figure 3F). As expected, Lineweaver-Burk plot analysis of the binding data reveals that TG53 competes for the same binding site in TG2 as FN42 (Figure 3F). A structurally similar compound incapable of blocking TG2-FN interaction as measured by AlphaLISA assay was selected from the ChemDiv library (TG288, see structure in Figure S1A in File S1) and used as a negative control. The ELISA signal was blocked by TG53, but not by TG288 (Figure S3B in File S1).

### Selected SMIs Block Cancer Cell Adhesion

**Figure 4:**
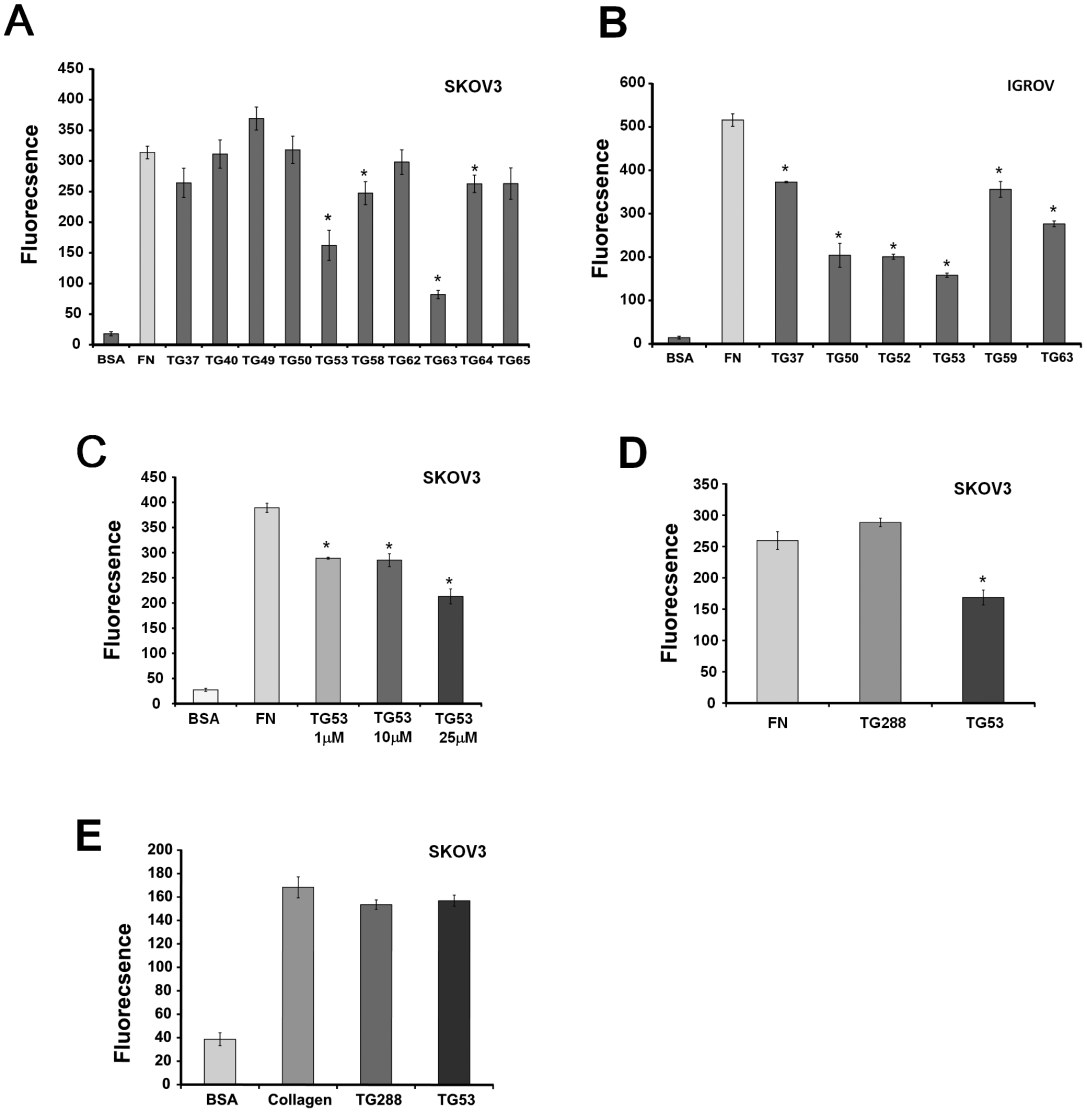
Top SMIs selected from the AlphaLISA based HTS inhibit cell adhesion to FN. **A**, Effects of top compounds (25 μM) on SKOV3 cells adhesion to wells coated by 5 μg/mL of FN. **B**, Effects of top compounds (25 μM) on IGROV1 cells adhesion to FN. **C**, Dose-dependent effects of compound TG53 (1–25 μM) on SKOV3 cells adhesion to FN. **D**, Comparison between TG53 (25 μM) and a structurally similar, but inactive compound (TG288, 25 μM), on SKOV3 cells adhesion. **E**, Effects of TG53 (25 μM) on SKOV3 cells adhesion to wells coated with collagen type I (20 µg/ml). Bars represent means +/− s.e.m. of quadruplicate measurements. Asterisks denote *p <*0.05.

As TG2-FN interaction critically regulates cellular adhesion to the ECM, we next studied the effects of selected SMIs on cell adhesion to FN by using SKOV3 and IGROV1 ovarian cancer cells that endogenously express TG2. We had previously demonstrated using these cells that stable TG2 knock-down inhibited adhesion to FN [5]. For this, we used a solid phase adhesion assay in the presence of the selected compounds or vehicle (control). TG53, TG58, TG63 and TG64 significantly inhibited cell adhesion to FN in SKOV3 cells (Figure 4A). Similar effects were noted in IGROV1 cells (Figure 4B); of the selected SMIs, TG53 showing consistent and >50% inhibition in both cell lines. Subsequent characterization of TG53 demonstrated dose-dependent inhibition of cell adhesion to FN (Figure 4C), compared to structurally similar, but inactive TG288 (Figure 4D). Furthermore, TG53 did not inhibit cell adhesion to collagen, another ECM protein, suggesting specificity to the TG2-FN complex (Figure 4E).

### TG53 Blocks Cell Migration and Invasion

**Figure 5:**
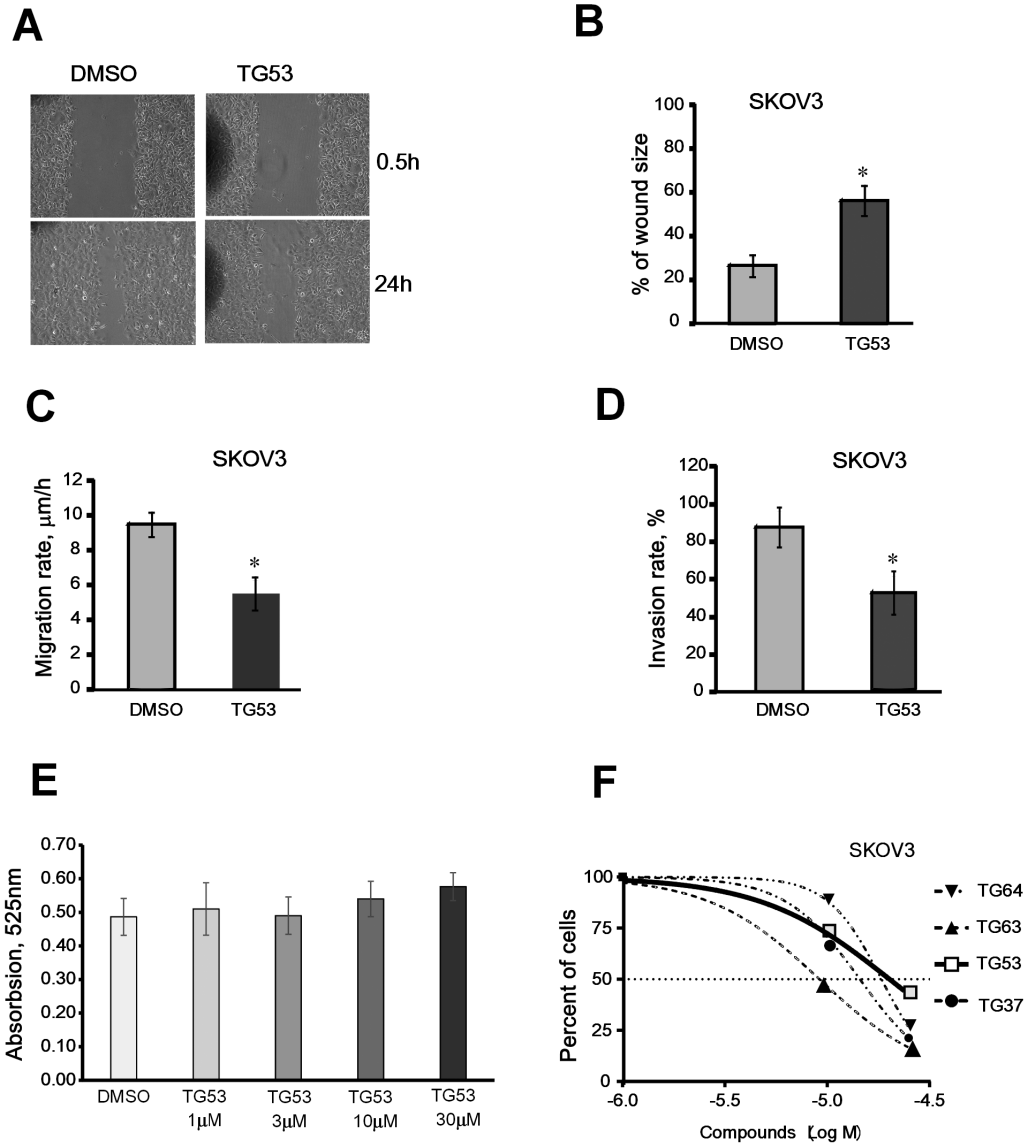
Effect of TG53 on SKOV3 cell migration, invasion, and proliferation. **A**, Representative phase-contrast microscopy images of cells migrating into the wounded area in an *in vitro* scratch wound healing assay. SKOV3 cells incubated in serum-free media supplemented with 1% DMSO or TG53 diluted to 10 μM in DMSO at time 0 (0.5 h) and 24 hours after wounding. **B**, Wound closure was assessed as the distance between the wound edges, calculated and quantified using Image Pro-Express software 4.01. The percentage of wound closure was evaluated using the formula (wound size at 24 hrs/ initial wound size) × 100. Data are shown as means ± SD of triplicate experiments. **C**, Cell migration rate (μm/h) was quantified as the difference between wound edges at 0 and 24 h, divided by 24. Results are means ± SD of three independent experiments. **D**, Numbers of SKOV3 cells invading through matrigel coated transwells were counted in 5 random fields. Results are means ± SD of duplicate experiments. **E**, Effects of TG53 (1–30 μM) on TGase activity, measured as described and compared to control (DMSO). **F**, Effects of top inhibitors selected from the AlphaLISA on SKOV3 cell proliferation measured by CCK-8 assay. Dose-response curves representing percentage of surviving cells were plotted using GraphPad Prism against the logarithmic concentrations of drugs used during 48 h treatment. Four SMIs were tested: TG37, TG53, TG63 and TG64.

As cell adhesion and migration are intimately linked, we next explored the effects of TG53 on cell motility and invasiveness by using the wound healing and the transwell invasion assays. Changes in wound size (distance between wound edges) at 24 hours post wounding and the rate of cell migration were analyzed, as described. TG53 diluted to 10 μM significantly decreased wound closure (Figure 5A–B; p<0.05) and cell migration rate (p<0.05; Figure 5C) as compared to DMSO control. Furthermore, the invasion rate through a trans-well coated with matrigel was also decreased by TG53 compared to control (Figure 5D, p<0.05), supporting that the compound inhibits cell motility and invasiveness.

### TG53 Inhibits Cell Proliferation without Affecting TG2 Enzyme Activity

As the catalytic domain is distinct from the FN-binding region of TG2, we did not expect that SMIs that interfere with the TG2-FN interaction would alter the transamidating activity of TG2. Indeed, TG53 did not inhibit TG2 enzymatic activity at 1–30 μM concentrations (Figure 5E). Anti-proliferative properties of selected compounds, including those of TG53, were characterized by using a CKK-8 assay after 48 hour incubation in the presence of compounds diluted from 1 to 100 μM or vehicle. Dose-response analyses revealed EC_50_ values in the 9 μM (e.g. TG63) to 21 μM range (e.g. TG53; Figure 5F). TG53 induced less cytotoxicity compared to other SMIs selected from the screen (e.g. TG64, TG63, and TG37), suggesting less off-target effects; however the compound was toxic to ovarian cancer cells at higher concentrations. Importantly, TG53 was not cytotoxic at concentrations up to 30 μM during shorter periods of treatment (Figure S4 in File S1), as used in the previously described bioassays.

## Discussion

In this manuscript, we describe a new strategy to measure the interaction between TG2 and FN through proximity based binding AlphaLISA assay [22]. Application of this assay to the ChemDiv library of chemical compounds led to the discovery of several potent TG2-FN inhibitors, which were subsequently shown to block cell adhesion and migration in OC cell lines. Our findings have several implications.

First, we demonstrate that the TG2-FN interaction is druggable. Generally PPIs represent a daunting target for disruption by small molecules due to their large interfacial areas and their often noncontiguous contact points. However, the TG2-FN interaction is characterized by a TG2 β-hairpin inserting into a deep pocket of FN, rendering it an attractive complex to be disrupted by SMIs. Resolution of the three-dimensional structure of TG2 has provided important insight into the complex regulation of its functions [18], [23], [24]. In addition to the catalytic triad consisting of Cys- 277, His-335, and Asp-358, the protein has the FN-binding within its N-terminal. The binding site to FN is located around the β-hairpin loop (amino acids 88–106), and mutations within this sequence significantly alter formation of a complex with FN [1].

On the surface of various cells, the complex between TG2 and FN is further supported and stabilized by direct interactions of both proteins with integrins, the major adhesion receptors involved in cellular adhesion to the ECM proteins, including FN. Due to modest affinity for the integrin-FN binding and strong noncovalent association of TG2 with both these proteins, TG2 significantly enhances the interaction of cells with FN serving as a bridge between integrins and FN, and a key mediator of the integrin-TG2-FN ternary adhesion complexes [4], [14]. Precise mapping of the integrin-TG2 interaction is difficult, as the composite integrin binding site on TG2 involves both its first and fourth domains, whereas the TG2-binding site on integrins includes several membrane-proximal epidermal growth factor (EGF) like repeats of the β subunit away from the FN-binding site (A.M. Belkin, unpublished results). While little is known about specifics of the integrin-TG2 complexes, the complementary TG2-FN binding sites have been delineated, and disruption of this interaction appears as promising approach for interfering with cell-ECM adhesion [14].

We have previously employed computational screening to identify small molecules that fit in the TG2-FN pocket; however, their specific binding to TG2 was difficult to demonstrate and the biological effects of the top identified hit in experiments evaluating cell adhesion remained modest [25]. Here we developed a robust assay using AlphaLISA technology [22] measuring the TG2-FN interaction that was applied to HTS of 10,000 chemical compounds. The assay has a Z′-factor of 0.7 and identified in robot-assisted screening 90 primary hits; of which 14 were consequently validated. It is anticipated that more SMIs and/or SMIs with improved inhibitory activity will be discovered by using this assay in a larger collection of compounds. Importantly, several of the selected hits demonstrated biological activity in subsequent bio-assays (e.g. inhibited cell adhesion to matrix proteins and cell migration), suggesting that inhibition of the TG2-FN complex formation by the discovered SMIs had measurable functional consequences. The top hits belonged to two structurally unrelated classes of compounds that phenocopied each other, suggesting that the compounds may be specific for the TG2-FN interface. We recognize potential limitations of assays relying on purified labeled proteins that might differ from their native conformations. The specific binding of selected SMIs to the TG2-FN pocket remains to be demonstrated in future analyses.

One of the newly discovered SMIs, TG53, was subsequently characterized through complementary assays including dose-response analyses, ELISA measuring the TG2-FN interaction, and other bioassays quantifying cell adhesion, migration, and proliferation. The focus on TG53 was based on its predicted drug-like properties and highest observed inhibitory activity in cell-based assays (e.g. cell adhesion) compared to the other compounds. TG53 performed optimal among selected hits in an ELISA measuring the TG2-FN interaction. Comparison to the effects of an inactive, but structurally similar compound (TG288) suggests specificity to the TG2-FN complex. However, we cannot exclude off target effects, particularly since the agent displays modest cytotoxic effects at high concentrations.

The drug-like potential of TG53 is supported by its predicted physico-chemical properties computed based on its chemical structure by using the ADMET Predictor and ChemSpider software. It is encouraging that TG53 is predicted to be orally bioavailable, violates none of the Lipinski’s rule of five, and passes the Muegge filter, suggesting that it can serve as a suitable basis for the development of drug like agents [26]. The LogP value of TG53 was 4.5; well within the range of most SMIs approved for clinical use and its aqueous solubility was low at 5.45 mole/liter. Its predicted cellular permeability in Madin-Darby canine kidney (MDCK) cells was 378 [27], the highest among the top hits selected from the screen, indicating intermediate solubility. Further optimization of this compound may increase its potency, cellular permeability, and bioavailability.

As the TG2-FN interaction plays a role in cell adhesion to the matrix, we postulate that SMIs targeting this complex may be developed into agents that block cancer metastasis, particularly for tumors like ovarian cancer that rely on adhesion to the ECM, as a primary mode of dissemination. We recognize that SMIs targeting the TG2-FN interaction might interfere with other physiological processes mediated by this PPI, such as formation of blood clots, wound healing, or certain immune responses involving cell adhesion to FN [28]–[31]. Therefore, future evaluation of such SMI *in vivo* must include careful assessment of potential toxic effects due to interference with TG2-mediated physiologic processes.

In summary, our results support that the TG2-FN interaction is a novel targetable PPI whose disruption could inhibit cell adhesion to the ECM. The AlphaLISA^TM^ technology based assay developed here is suitable for HTS and can be used to screen larger libraries. We propose that the top compound identified, TG53, is a specific inhibitor of the TG2-FN complex with potential utility as a novel therapeutic targeting cancer metastasis or as a new biochemical tool to study cell adhesion to the matrix.

## Supporting Information

File S1 **Figures S1–S4.**
(DOCX)

## References

[1] Hang J, Zemskov EA, Lorand L, Belkin AM (2005) Identification of a novel recognition sequence for fibronectin within the NH2-terminal beta-sandwich domain of tissue transglutaminase. J Biol Chem 280: 23675–23683. 15849356 10.1074/jbc.M503323200

[2] Akimov SS, Belkin AM (2001) Cell-surface transglutaminase promotes fibronectin assembly via interaction with the gelatin-binding domain of fibronectin: a role in TGFbeta-dependent matrix deposition. J Cell Sci 114: 2989–3000. 11686302 10.1242/jcs.114.16.2989

[3] Radek JT, Jeong JM, Murthy SN, Ingham KC, Lorand L (1993) Affinity of human erythrocyte transglutaminase for a 42-kDa gelatin-binding fragment of human plasma fibronectin. Proc Natl Acad Sci U S A 90: 3152–3156. 8097314 10.1073/pnas.90.8.3152 PMC46257

[4] Zemskov EA, Janiak A, Hang J, Waghray A, Belkin AM (2006) The role of tissue transglutaminase in cell-matrix interactions. Front Biosci 11: 1057–1076. 16146797 10.2741/1863

[5] Satpathy M, Cao L, Pincheira R, Emerson R, Bigsby R, et al (2007) Enhanced peritoneal ovarian tumor dissemination by tissue transglutaminase. Cancer Res 67: 7194–7202. 17671187 10.1158/0008-5472.CAN-07-0307

[6] Verma A, Wang H, Manavathi B, Fok JY, Mann AP, et al (2006) Increased expression of tissue transglutaminase in pancreatic ductal adenocarcinoma and its implications in drug resistance and metastasis. Cancer Res 66: 10525–10533. 17079475 10.1158/0008-5472.CAN-06-2387

[7] Mann AP, Verma A, Sethi G, Manavathi B, Wang H, et al (2006) Overexpression of Tissue Transglutaminase Leads to Constitutive Activation of Nuclear Factor-{kappa}B in Cancer Cells: Delineation of a Novel Pathway. Cancer Res 66: 8788–8795. 16951195 10.1158/0008-5472.CAN-06-1457

[8] Kumar A, Xu J, Brady S, Gao H, Yu D, et al (2010) Tissue transglutaminase promotes drug resistance and invasion by inducing mesenchymal transition in mammary epithelial cells. PLoS One 5: e13390. 20967228 10.1371/journal.pone.0013390 PMC2953521

[9] Verma A, Guha S, Diagaradjane P, Kunnumakkara AB, Sanguino AM, et al (2008) Therapeutic significance of elevated tissue transglutaminase expression in pancreatic cancer. Clin Cancer Res 14: 2476–2483. 18413840 10.1158/1078-0432.CCR-07-4529

[10] Satpathy M, Shao M, Emerson R, Donner DB, Matei D (2009) Tissue transglutaminase regulates matrix metalloproteinase-2 in ovarian cancer by modulating cAMP-response element-binding protein activity. The Journal of biological chemistry 284: 15390–15399. 19324884 10.1074/jbc.M808331200 PMC2708835

[11] Shao M, Cao L, Shen C, Satpathy M, Chelladurai B, et al (2009) Epithelial-to-mesenchymal transition and ovarian tumor progression induced by tissue transglutaminase. Cancer Res 69: 9192–9201. 19951993 10.1158/0008-5472.CAN-09-1257

[12] Cao L, Shao M, Schilder J, Guise T, Mohammad KS, et al (2012) Tissue transglutaminase links TGF-beta, epithelial to mesenchymal transition and a stem cell phenotype in ovarian cancer. Oncogene 31: 2521–2534. 21963846 10.1038/onc.2011.429

[13] Condello S, Cao L, Matei D (2013) Tissue transglutaminase regulates beta-catenin signaling through a c-Src-dependent mechanism. FASEB J. 10.1096/fj.12-222620 PMC4050431 23640056

[14] Akimov SS, Krylov D, Fleischman LF, Belkin AM (2000) Tissue transglutaminase is an integrin-binding adhesion coreceptor for fibronectin. J Cell Biol 148: 825–838. 10684262 10.1083/jcb.148.4.825 PMC2169362

[15] Herman JF, Mangala LS, Mehta K (2006) Implications of increased tissue transglutaminase (TG2) expression in drug-resistant breast cancer (MCF-7) cells. Oncogene 25: 3049–3058. 16449978 10.1038/sj.onc.1209324

[16] Fok JY, Ekmekcioglu S, Mehta K (2006) Implications of tissue transglutaminase expression in malignant melanoma. Mol Cancer Ther 5: 1493–1503. 16818508 10.1158/1535-7163.MCT-06-0083

[17] Yuan L, Siegel M, Choi K, Khosla C, Miller CR, et al (2007) Transglutaminase 2 inhibitor, KCC009, disrupts fibronectin assembly in the extracellular matrix and sensitizes orthotopic glioblastomas to chemotherapy. Oncogene 26: 2563–2573. 17099729 10.1038/sj.onc.1210048

[18] Liu S, Cerione RA, Clardy J (2002) Structural basis for the guanine nucleotide-binding activity of tissue transglutaminase and its regulation of transamidation activity. Proc Natl Acad Sci U S A 99: 2743–2747. 11867708 10.1073/pnas.042454899 PMC122418

[19] Borsi L, Castellani P, Balza E, Siri A, Pellecchia C, et al (1986) Large-scale procedure for the purification of fibronectin domains. Anal Biochem 155: 335–345. 3728983 10.1016/0003-2697(86)90443-4

[20] Zhang JH, Chung TD, Oldenburg KR (1999) A Simple Statistical Parameter for Use in Evaluation and Validation of High Throughput Screening Assays. J Biomol Screen 4: 67–73. 10838414 10.1177/108705719900400206

[21] Folk JE, Cole PW (1966) Transglutaminase: mechanistic features of the active site as determined by kinetic and inhibitor studies. Biochim Biophys Acta 122: 244–264. 5969301 10.1016/0926-6593(66)90066-x

[22] Pfluger M, Kapuscik A, Lucas R, Koppensteiner A, Katzlinger M, et al (2013) A combined impedance and AlphaLISA-based approach to identify anti-inflammatory and barrier-protective compounds in human endothelium. J Biomol Screen 18: 67–74. 22941294 10.1177/1087057112458316

[23] Han BG, Cho JW, Cho YD, Jeong KC, Kim SY, et al (2010) Crystal structure of human transglutaminase 2 in complex with adenosine triphosphate. International Journal of Biological Macromolecules 47: 190–195. 20450932 10.1016/j.ijbiomac.2010.04.023

[24] Pinkas DM, Strop P, Brunger AT, Khosla C (2007) Transglutaminase 2 undergoes a large conformational change upon activation. Plos Biology 5: 2788–2796. 10.1371/journal.pbio.0050327 PMC2140088 18092889

[25] Khanna M, Chelladurai B, Gavini A, Li L, Shao M, et al (2011) Targeting ovarian tumor cell adhesion mediated by tissue transglutaminase. Molecular cancer therapeutics 10: 626–636. 21330459 10.1158/1535-7163.MCT-10-0912

[26] Muegge I, Heald SL, Brittelli D (2001) Simple selection criteria for drug-like chemical matter. J Med Chem 44: 1841–1846. 11384230 10.1021/jm015507e

[27] Gertz M, Harrison A, Houston JB, Galetin A (2010) Prediction of human intestinal first-pass metabolism of 25 CYP3A substrates from in vitro clearance and permeability data. Drug Metab Dispos 38: 1147–1158. 20368326 10.1124/dmd.110.032649

[28] Belkin AM, Tsurupa G, Zemskov E, Veklich Y, Weisel JW, et al.. (2005) Transglutaminase-mediated oligomerization of the fibrin(ogen) {alpha}C-domains promotes integrin-dependent cell adhesion and signaling. Blood. 10.1182/blood-2004-10-4089 PMC1895018 15637140

[29] Belkin AM, Zemskov EA, Hang J, Akimov SS, Sikora S, et al (2004) Cell-surface-associated tissue transglutaminase is a target of MMP-2 proteolysis. Biochemistry 43: 11760–11769. 15362860 10.1021/bi049266z

[30] Falasca L, Iadevaia V, Ciccosanti F, Melino G, Serafino A, et al (2005) Transglutaminase type II is a key element in the regulation of the anti-inflammatory response elicited by apoptotic cell engulfment. J Immunol 174: 7330–7340. 15905580 10.4049/jimmunol.174.11.7330

[31] Fesus L, Piacentini M (2002) Transglutaminase 2: an enigmatic enzyme with diverse functions. Trends Biochem Sci 27: 534–539. 12368090 10.1016/s0968-0004(02)02182-5

