# Supplementary material for: Small Molecule Inhibitors Target the Tissue Transglutaminase and Fibronectin Interaction
Source: PLoS One. 2014 Feb 20;9(2):e89285. doi: 10.1371/journal.pone.0089285 (PMC3930694; doi:10.1371/journal.pone.0089285)
Supplement: File S1 — Figures S1–S4. (DOCX) [file pone.0089285.s001.docx]

**Supporting Information**

**:**


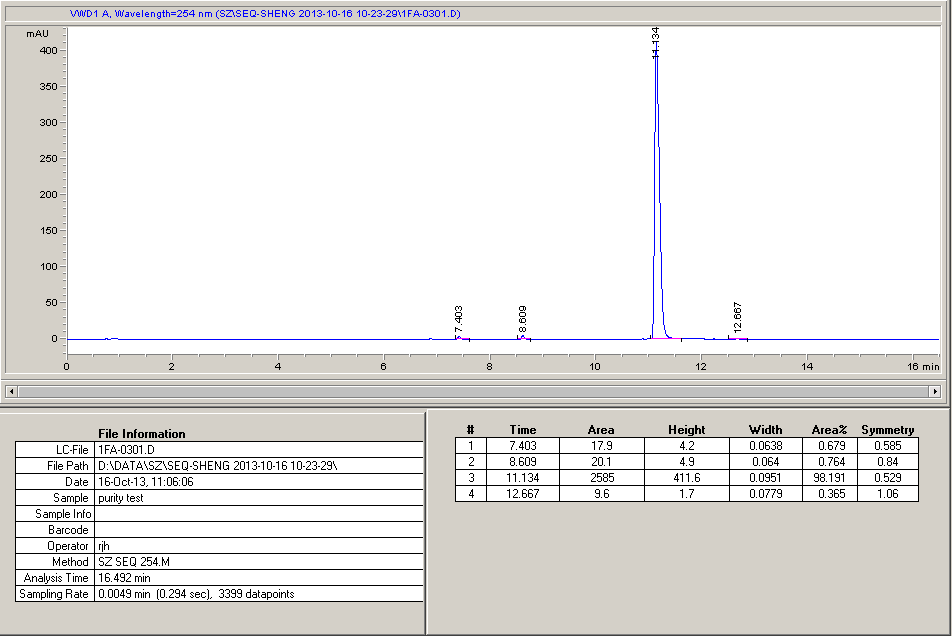


**Figure S1**

**Legend:** *HPLC-MS analysis of Compound* ***TG53***: tR = 11.1 min, purity = 98.2% (UV, λ = 254 nm). MS (ESI): calculated for [M], 411, found [M+H]^+^ 412. Analytical HPLC-MS analysis was carried out on an Agilent 1200 analytic HPLC system with a 6130 Quadrupole MS detector (see Material and Methods).

**TG37**

**TG40**

**TG49**

**TG50**

**TG52**

**TG53**

**TG57**

**TG58**

**TG62**

**TG63**

**TG64**

**TG65**

**TG67**

**Figure S2**

**Legend:** MS spectra of selected compounds tested in various experiments.

**A**

**

**

**B**

**

**

**Figure S3**

**Legend: A.** Structure of TG53 compared to that of the inactive compound TG288. **B.** ELISA measured dose dependent inhibition of TG2-FN42 interaction by TG53 and the negative control (TG288). Bars represent means +/- SD of triplicate measurements. Asterisks denote *p <*0.05.

**Figure S4**

**Legend:** Effects of various concentrations of TG53 on SKOV3 cell proliferation measured by CCK-8 assay measured at 1h (**A**), 4h (**B**), 8h (**C**), 24h (**D**).
